# The Influence of Clay Structures to the Hygrothermal Component of the Indoor Environment

**DOI:** 10.3390/ma15051744

**Published:** 2022-02-25

**Authors:** Jakub Diviš, Jan Růžička

**Affiliations:** 1 University Centre for Energy Efficient Buildings, Czech Technical University in Prague, 273 43 Bustehrad, Czech Republic; 2 Faculty of Civil Engineering, Czech Technical University in Prague, 166 29 Prague, Czech Republic; jan.ruzicka@fsv.cvut.cz

**Keywords:** dynamic sorption, moisture buffering, relative humidity, indoor environment, building materials, building structures, clay, rammed earth

## Abstract

In this article, research on the sorption properties of clay materials in comparison with commonly used building materials is published. The topic is mainly focused on the dynamic sorption properties and their influence on the relative humidity in the indoor environment. The results of comparisons of clay structures, rammed earth panels, clay plaster, and unburned bricks, with commonly used building materials, concrete, lime plaster, and gypsum board are examined. Statistically evaluated results in the form of confidence intervals are presented and the rate of dynamic sorption is analyzed. It is clear from the results that clay materials have a positive effect on the rapid adsorption and desorption of air moisture in the interior of buildings. However, there are many variables, band not every clay material has such excellent sorption properties.

## 1. Introduction

### 1.1. Indoor Air Quality

Nowadays, people spend almost 90% of their time in residential buildings, kindergartens, schools, offices, and other building facilities [1]. For this reason, it is also necessary to focus more on the quality of the indoor environment. Indoor air quality (IAQ) depends on many factors: outdoor air quality, amount of air pollutants, ventilation air volume, and ventilation system. In most cases, the air quality in buildings is worse than the air quality in the outdoor environment. Deteriorated ambient air quality is primarily the result of energy consumption in transportation and industry and in building construction. Pollutants associated with the operation of buildings account for approximately 40% of total pollutant production, of which ventilation accounts for up to 50% [2].

In the climate of Central Europe, building ventilation requires air treatment for most of the year: heating, cooling, humidification, and dehumidification. Forced ventilation can effectively maintain the required indoor climate quality in buildings. However, these active indoor climate control systems are energy intensive. The total energy consumed in this way will cause a further increase in external pollution. Outdoor pollution can have various impacts depending on the extent to which it is reported: regional (SO_2_, NO_x_) or global (CO_2_) [3].

From an environmental point of view, it is therefore better to replace active control systems with passive ones.

#### 1.1.1. Indoor Microclimate

A building environment created for human occupation in enclosed spaces can be characterized as an indoor microclimate. It can be divided into the following constituents: hygrothermal microclimate, air quality (odor, toxic, aerosol, microbial), ionizing, electrostatic, electromagnetic, electronic, acoustic, lighting, and psychological microclimate. The organisation CPD (Centre for Passive House) describes the importance of each component [4].

The hygrothermal microclimate is a component of the environment formed by heat and humidity fluxes. The optimal thermal-humidity state of the indoor environment is important not only for human health but also for the proper functioning of the building itself [5].

#### 1.1.2. Relative Humidity in Buildings

Relative indoor humidity (RH) is one of the crucial indicators of the internal microclimate quality. It is defined as the ratio of the partial pressure (or density) of water vapour in the air to the saturated partial pressure (or density) of water vapour at a given temperature and same total pressure [6].

Relative humidity in the interior of a building changes over time. It depends mainly on the intensity of ventilation, the relative humidity of the exterior, the interior temperature, and the internal sources of water vapour. In typical dwellings, the main indoor sources of moisture are human activities (cooking, showering, washing, ironing, etc.), water vapour production from human respiration, and plant transpiration. The average dwelling produces 4–15 kg of water vapour per day [7,8].

In summer (for the Central European inland region, temperate climate), the water vapour content of outdoor air is high. After such warm air is brought into the building interior and subsequently cooled. The resulting air is humid, and the RH can rise above 70%. In winter, the problem is reversed. There is a small amount of water vapour in the outdoor air. When such cold air is brought indoors and warmed (without further humidity treatment), the resulting air is dry, and the RH may fall below 30%. These extreme conditions bring their own risks [7].

The comfort zone for humans is described by the hygrothermal microclimate of the interior through a combination of air temperature and relative humidity. The optimum humidity of the internal environment fluctuates from 40 to 60%. Relative humidity in the range of 30 to 70% is still considered a comfortable indoor environment [7,9,10,11,12].

#### 1.1.3. Summary

From the above, it is clear that the value of relative humidity in the interior plays an important role in IAQ. Therefore, the properties of indoor air or air supply are currently being refined.

However, altering relative humidity in air-conditioning units is energy intensive and cost-consuming. This is not in line with the global push to reduce energy consumption [13] and increase energy efficiency in all aspects of life, including construction.

It should be noted that relative humidity can also be regulated, without any operating energy, by choosing suitable building structures and structural materials with moisture buffering properties. The use of porous building materials can be a passive indoor humidity control system. Porous materials respond to fluctuations in air humidity, which they can adsorb or desorb, thereby reducing extreme peaks and keeping the relative humidity level within the desired range. In addition, by using low-carbon porous building materials, we can achieve a lower carbon footprint when constructing or renovating buildings.

Clay materials, compared to other building materials, have both a low environmental impact [14,15,16] and a high specific surface area [17,18,19,20]. Therefore, it is a very suitable material for interior moisture control [21,22,23].

The following search deals specifically with the sorption properties of clay materials and structures; both steady state and dynamic.

### 1.2. Sorption Properties of Clay Materials and Structures

#### 1.2.1. Sorption Properties in Steady State

In many articles, significantly better sorption properties of clay materials compared to commonly used building materials as measured in terms of sorption isotherms have been presented [21,24,25,26,27,28,29,30,31,32,33].

The results clearly show that the higher amount of clay minerals in the clay materials, the higher the absorption capacity of the materials. This is further influenced by the content of various types of clayey minerals.

The hygrothermal properties of clay materials were also measured in the context of their chemical stabilization with cement or lime. This principle is used to ensure greater mechanical stability. However, it slightly degrades the sorption properties and the environmental impact.

The result of the search shows that a large number of experimentally measured sorption isotherms of clay materials can be traced, while individual results cannot be directly compared. The mixtures were often measured under different boundary conditions that directly affect the resulting values.

The type and amount of clay minerals used, the chemical stabilization, the ratio of clays and sands in the mixture, etc. have a fundamental effect on the sorption isotherms of clay materials.

The sorption isotherm is understood as a hygrothermal property of the material, but it itself has little informative value for construction methods. However, a significant application can be found in dynamic calculations (e.g., WUFI) and in mathematical zone moisture models. It is also part of the MBV calculation.

However, adsorption and desorption isotherms are measured only at a steady state. The comparison is inappropriate for realistic behaviour as the dynamic process of sorption of indoor air moisture is not reflected. The sorption isotherms also do not tell anything about the building structure itself, which typically consists of different materials in several layers. Therefore, the following is a search for the dynamic sorption properties of building structures, not only materials.

#### 1.2.2. Sorption Properties in Dynamic State

Already in his doctoral thesis [34], Carsten Rode Pedersen devoted himself to the hygrothermal behaviour of composite building structures and the theory of combined heat and moisture transport. The dynamic sorption properties of materials can be measured using various methodologies, e.g., Nordtest (MBV = moisture buffer value [35]) or Japanese JIS. The differences in the principles and measurement results of the two methodologies are also described here [36].

McGregor et al. (2014) in their article [33] presented the results of measuring the potential to regulate indoor humidity using various building materials using the moisture buffering value (MBV) concept. Measurements of MBV and steady state properties were performed on 18 samples of clay materials (compressed earth blocks and stabilised compressed earth blocks). It also showed how the variability of experimental conditions in the dynamic measurement can change the obtained MBV. The results of other articles [21] indicate that unfired clay material has a much greater potential to regulate indoor humidity than conventional construction materials previously reported in the literature.

Liuzzi et al. (2013) dealt with dynamic sorption properties and zone moisture modelling. The subject of the research [26] was various clay mixtures without chemical stabilization or with the addition of lime. Cascione et al. (2020) presented results [37] of the moisture buffering capacity of three hygroscopic plasters (clay, gypsum and lime) and compared them with the numerical simulations of a single-zone room space.

Other dynamic experiments of clay materials were performed by Maskell, D. Thomson, A., Walker, P., and Lemke, M. (2018) to determine the optimal plaster thickness for moisture buffering of indoor air [38]. In the case of a nonsteady state, the water molecules penetrate only to a certain depth of the building structure during dynamic adsorption and desorption. In this paper, it is demonstrated that there is a thickness of material beyond which there is no increase in moisture buffering capacity. The importance of determining the depth of moisture buffering penetration results in the possibility of optimizing the final layer structure (thickness, topcoats, etc.) for an effective internal moisture buffering performance.

#### 1.2.3. Summary

The search shows that the dynamic sorption properties were measured mainly for clay materials, not for whole building structures. In some cases, this may not correspond to the actual composition of the structure, which is usually composed of multiple materials. The aim of this research was to focus specifically on building structures and to investigate the moisture response of the indoor environment on the sorption properties of building structures during a step change in relative humidity in the interior. For the experimental verification of sorption properties, the methodology developed at the Faculty of Civil Engineering of the Czech Technical University in Prague was chosen [39]. The main idea of this applied dynamic sorption test is to simulate real situations in the building and the behaviour of real building structures under real-world conditions.

The trend in today’s construction industry is to reduce energy consumption while reducing associated and operational emissions. There is also an emphasis on the use of recyclable materials [13]. Clay materials offer all this.

## 2. Materials and Methods

The aim of the research was to describe the influence of the dynamic sorption properties of clay materials on the relative humidity of the indoor environment when the relative humidity changes. A medium-dimensional dynamic sorption test for building structures, developed by Diviš, Růžička (2016) [40], was used.

Two variants of the test are proposed in the methodology: desorption and adsorption dynamic test. In this paper, the influence of dynamic adsorption properties (i.e., variant II) of selected wall elements is compared and the moisture response of the indoor environment is investigated. Partial test results demonstrate the sorption potential of unfired clay structures (rammed earth, unfired clay brick, unfired clay brick with clay plaster) compared to commonly used building materials (concrete, ceramic brick with lime plaster, and plasterboard partition).

The Dynamic sorption properties experiment of real structural parts was performed at UCEEB CVUT in Prague, in a hygrothermal laboratory. The measured results are presented in the paper, including an uncertainty analysis and a comparison of selected wall elements.

### 2.1. Proposed Methodology for Medium-Scale Experimental Test

According to the methodology used, the following parameters were measured: temperature T [°C], relative humidity RH [%], and the amount of water consumed m [g]. Individual variables were measured in minute steps.

The following were monitored: the dynamic change of relative humidity in the environment, the amount of water supplied to the environment (moisture gain), and the ambient air temperature. All these factors directly affect the rate of dynamic sorption.

**Figure 1:**
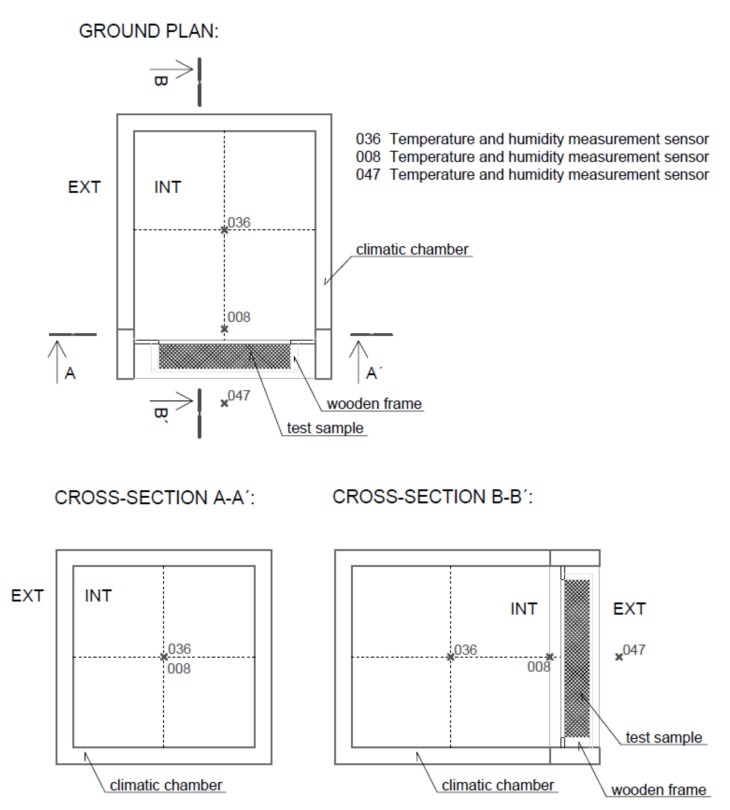
Diagram of the instrumentation scheme: climatic chamber; test sample; location of the sensors.

Relative humidity and temperature measurements were taken continuously at three locations: outside the chamber (laboratory), in the middle of the climate chamber, and on the front surface of the tested samples. Furthermore, the amount of water used by the equipment to maintain the set RH level in the climate chamber was measured (Figure 1).

The structural parts were tested in the climate chamber with the test space dimensions approx. 950 mm (height), 1100 mm (width), 950 mm (depth). The material of the inner surface of the test chamber is polished stainless steel.

The measuring apparatus:

- climate test chamber WEISS WK3-1000/0-S;
- laboratory balance AND GX-4000 for measuring water consumption;
- datataker DT85 Series 3 for data recording;
- temperature and humidity measurement sensors Rotronic HC2-S+E2-05XX.

**Figure 2:**
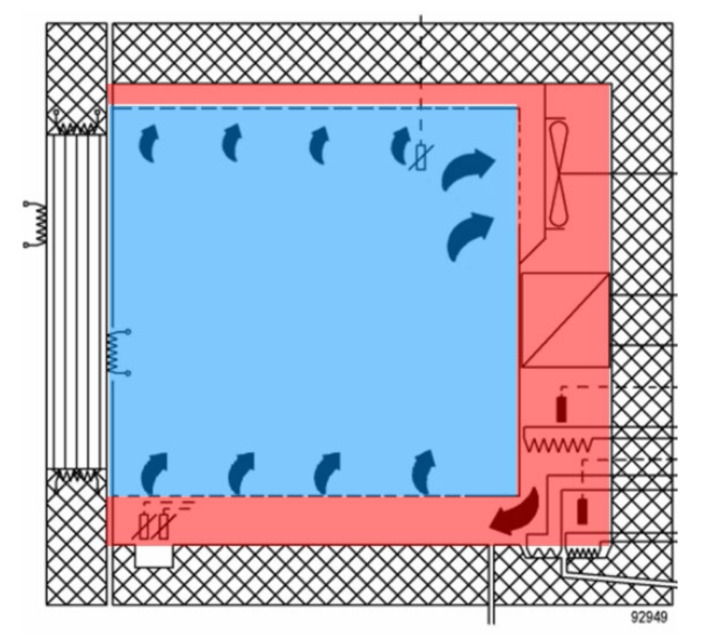
Cross-section of test chamber: usable space (blue); installation space (red).

The volume of the test chamber according to the manufacturer is approx. 993 litres. However, this is only the usable space; installation space is not included (Figure 2). For the experiments described below, it is necessary to know the entire volume of air that is regulated in the chamber. The total volume of the test space is V = 1.3 m^3^.

**Figure 3:**
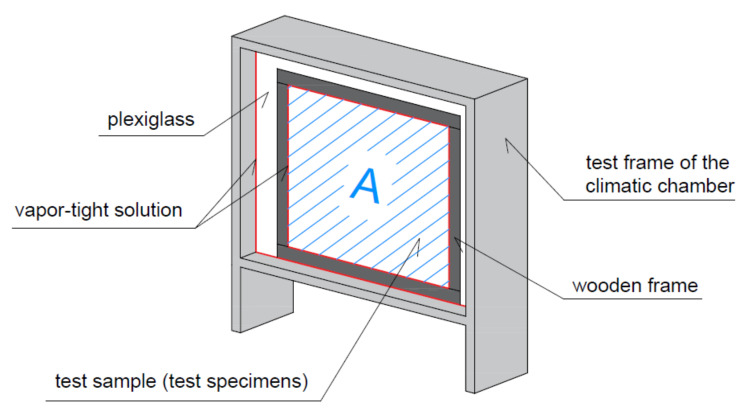
Vapour-tight solution: attaching the test specimen to the test frame.

The dynamic sorption properties of real structures were tested in the climatic chamber on specimens from structural parts. The dimension of the exposed area A (test specimen without wooden frame) of all test specimens was the same, 820 mm × 750 mm, that is, A = 0.615 m^2^ (Figure 3).

The A/V is the ratio between the exposed surface area of the test specimen and the total volume of the test chamber. The ratio was the same for all experimental structures, i.e., the A/V is 0.473 m^2^ m^−3^. Changing the ratio would change the results! The higher the ratio, the more the structure can adsorb/desorb air humidity from the environment. Conversely, the smaller the ratio, the smaller the amount of water vapour the structure can absorb from the air.

The fabricated test specimens were conditioned in laboratory conditions at 45 ± 10% RH and a laboratory temperature of 23 ± 2 °C for 2.5 months. Subsequently, the test sample was placed in a climatic chamber, where it was conditioned at a temperature of T = 23 °C, and a relative humidity of RH = 45%. This conditioning lasted 4 to 7 days. The whole test was isothermal (constant chamber temperature).

The proposed test method for climatic chamber is based on the following situation: the internal environment and the tested structure are in an equilibrium state, then the internal environment is faced with an intense increase of relative humidity for a certain period of time (i.e., someone is taking a shower or bath, cooking, drying laundry, etc.). At the end of the high humidity period, relative humidity in the climate chamber is monitored to determine the potential of the tested building structure to mitigate humidity peaks and absorb moisture from the environment (adsorption potential of the building structure).

**Figure 4:**
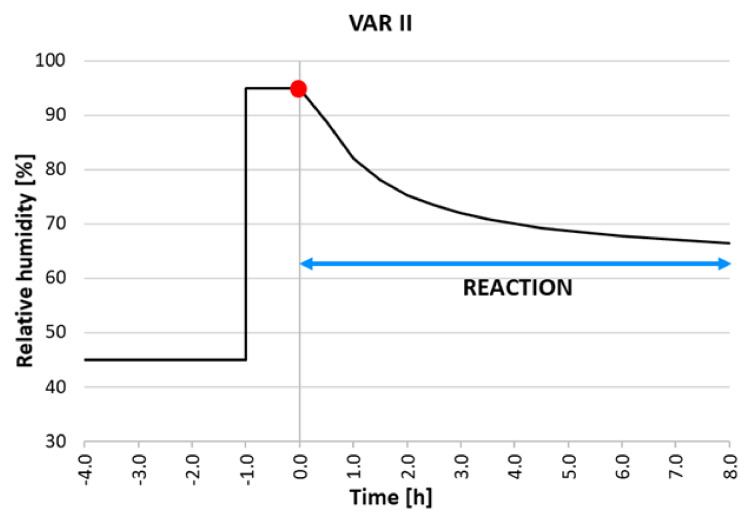
Scheme of the proposed methodology: dynamic adsorption test.

This test method was carried out at a constant temperature of 23 °C in the climatic chamber, and the testing of dynamic adsorption consisted of the following steps (also shown in Figure 4):

1. Conditioning of the test sample and the internal environment to an equilibrium state at T = 23 °C and RH = 45% for 48 h;
2. Fast increasing of relative humidity at 95% RH for 60 min;
3. Monitoring of RH changes in the climatic chamber for 8 h.

### 2.2. Materials and Building Structures

The test specimens were installed in a wooden frame with the rear side covered by OSB board 12 mm thick. The rear and perimeter sides of the test samples were covered with Gutta PE foil (Rotkreuz, Switzerland) to avoid side moisture transfer. The joints were sealed with aluminium adhesive tape and ISOCELL AIRSTOP SPRINT sealant (Neumarkt am Wallersee, Germany). The samples produced in this way were inserted into the test frame of the climatic chamber.

The rest of the test space between the sample and the steel frame of the test chamber was filled with 6 mm thick plexiglass. This space was necessary for the installation of the sample. The vapour-tight connection of the plexiglass and the steel frame was secured by Terostat-IX sealant (Düsseldorf, Germany), the connection of the PE foil from the sides of the sample and the plexiglass was ensured by ISOCELL AIRSTOP SPRINT sealant (Neumarkt am Wallersee, Germany) and aluminium adhesive tape or ISOCELL AIRSTOP FLEX adhesive tape (Neumarkt am Wallersee, Germany).

These vapour barrier measures ensured the transmission of water vapour only in the 1D direction between the surface of the test sample *A* and the volume of the climatic chamber *V*. Figure 2 shows the fitting of the test sample in the chamber test frame and the execution of the vapour barrier connection.

**Figure 5:**
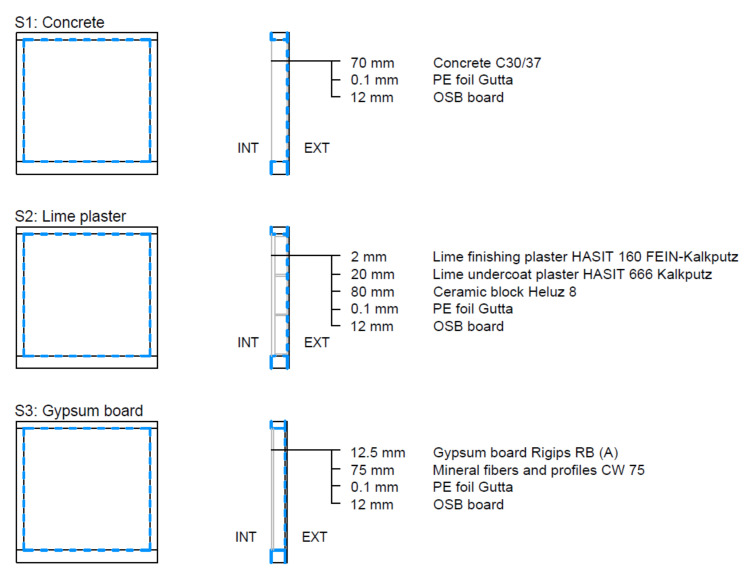
Traditional wall elements: test samples S1–S3.

**Figure 6:**
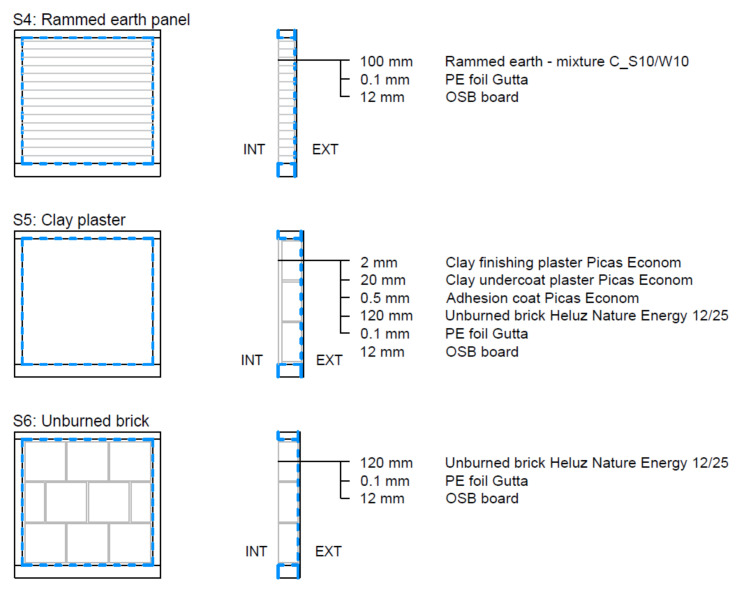
Clay-based wall elements: test samples S4–S6.

**Figure 7:**
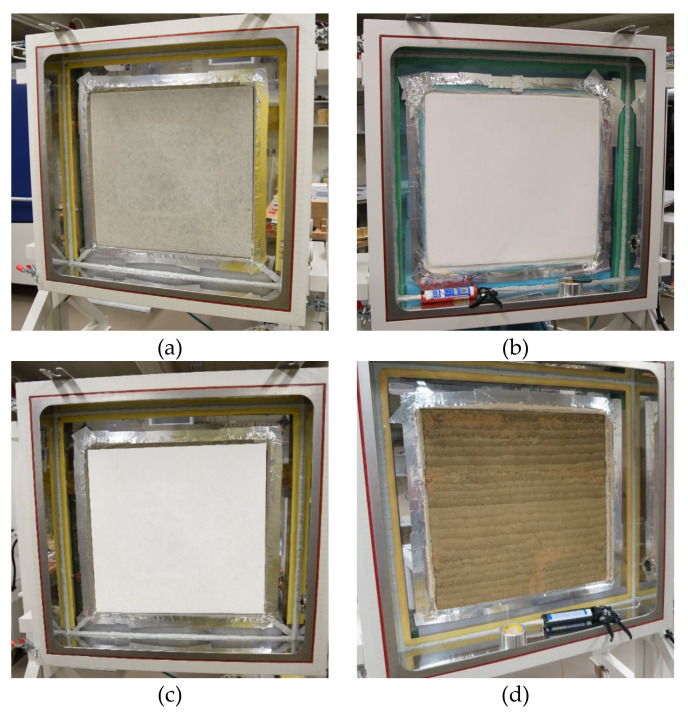

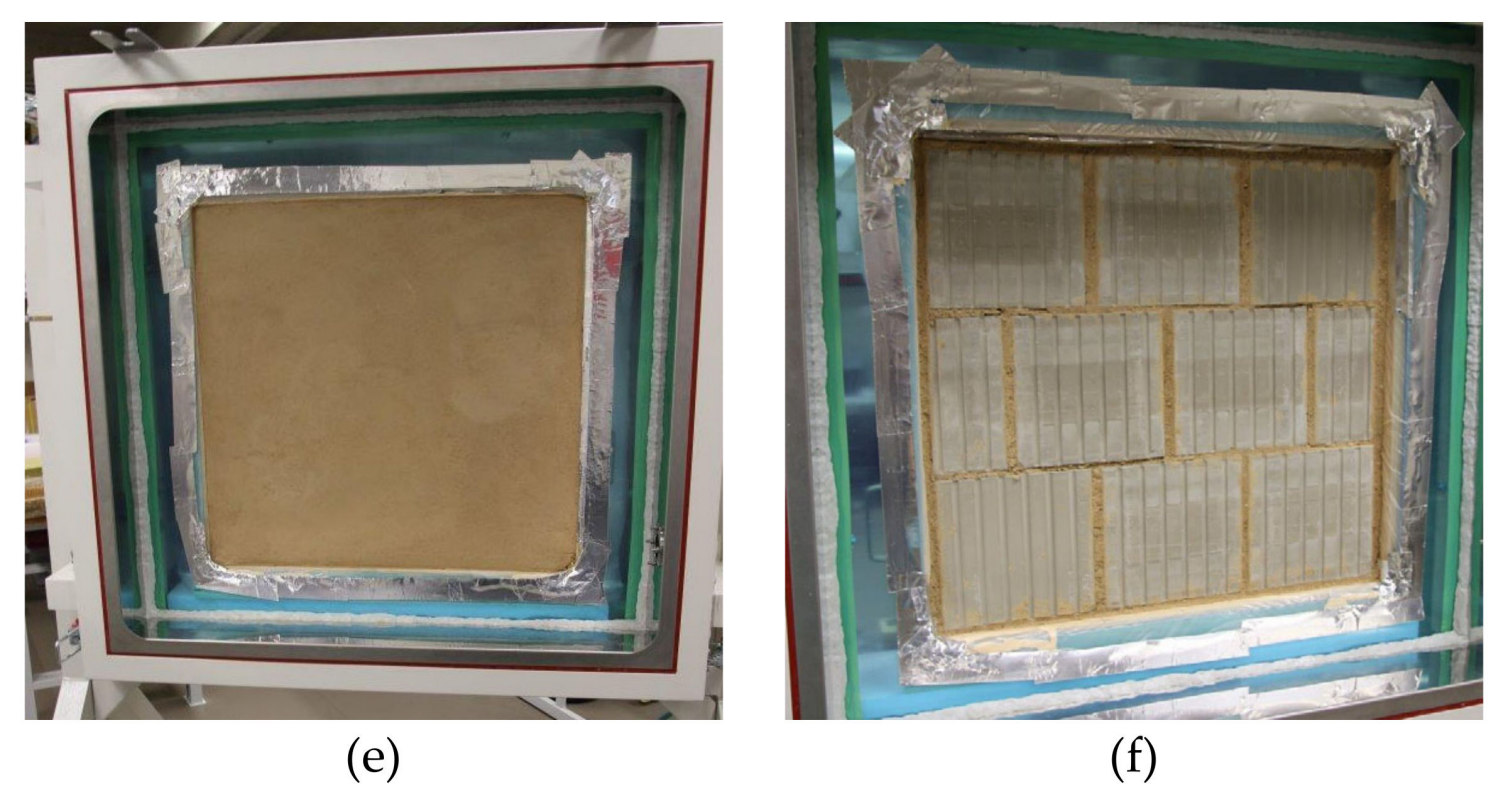
Testing wall elements in the climatic chamber: (**a**) concrete wall; (**b**) ceramic hollow blocks with lime plaster; (**c**) partition wall from gypsum board; (**d**) rammed earth panel (C_S10/W10); (**e**) unburned clay hollow blocks with clay plaster; (**f**) fair-face brickwork from unburned clay hollow blocks.

The following test samples were tested: plexi board, concrete, lime plaster, gypsum board, rammed earth panel, clay plaster, and unburned brick (Figure 5, Figure 6 and Figure 7).

**Plexi board**

Plexiglass board was a reference material. However, the structure was mainly used to verify the experiment, because the material is diffusion-impermeable, and the airtightness of the experimental measuring device and apparatus can be verified with it. The material was used: plexiglass XT (Röhm GmbH, Darmstadt, Germany), extruded, thickness 6 mm, 6/000/XT.

**Concrete**

Structure S1 was a mixture of common concrete C30/37. This mixture consisted of cement CEM I 42.5 R (Českomoravský Cement, a.s., Mokrá-Horákov, Czech Republic), aggregate fraction 4–16 mm, aggregate fraction 0–4 mm (KÁMEN Zbraslav, a.s., Zbraslav, Czech Republic), and water. The construction thickness was 70 mm.

**Lime plaster**

Structure S2 was made of ceramic hollow blocks Heluz 8 (Heluz, Dolní Bukovsko, Czech Republic) of thickness 80 mm. The masonry mortar was from Cemix 5 (Cemix, Pyskowice, Poland). The structure was covered by lime plaster HASIT 160 Fein Kalkputz (Horažďovice, Czech Republic), without additional surface treatment (coating). The total thickness was 22 mm (20 + 2).

**Gypsum board**

Structure S3 was a partition wall, it was made by gypsum board Rigips RB (A) (Gelsenkirchen, Germany) thickness 12.5 mm without surface treatment (coating). The supporting structure consisted of a metal stud CW 75, which was filled with thermal insulation of mineral fibers.

**Rammed earth panel**

Structure S4 was made of rammed earth. The mixture C_S10/W10 was made of a mixture of raw clay material from the Czech company Claygar (Olomouc, Czech Republic) (90%), sand of fraction 0–4 (10%), and water (10%). The height of the compacted layers was 40–50 mm. The rammed earth panel was 100 mm thick. This mixture was designed within the framework of development at the CTU in Prague [41].

**Clay plaster**

Structure S5 was made of unburned clay hollow blocs Heluz Nature Energy 12/25 (Dolní Bukovsko, Czech Republic), of 120 mm thick. The masonry mortar was from the clay mixture Picas Econom (Hradčany, Czech Republic). The surface layer was made of clay plaster PICASS ECONOM (Hradčany, Czech Republic) with total thickness of 22 mm (10 + 10 + 2).

**Unburned brick**

Structure S6 was made of fair-face brickwork from unburned clay hollow blocks Heluz Nature Energy 12/25 (Dolní Bukovsko, Czech Republic), 120 mm thick. The masonry mortar was from the clay mixture Picas Econom (Hradčany, Czech Republic).

### 2.3. Methodology for Evaluation of Measured Data of Dynamic Sorption Properties of Building Structures

Experimental measurements on each structure were performed three times. The main reason for this was the time and economic demands of the experiment. Although a higher number of measurements would have reduced the measurement uncertainties, three repetitions is a sufficient set to determine the measurement uncertainties.

Statistical evaluation of the dynamic sorption properties measured on six wall elements was performed according to the proposed methodology. For the evaluation, the following steps had to be performed:

- In the first step, it was necessary to smooth individual observations using regression analysis. The suitable regression curves were determined using the least squares method. Regression parameters were determined for polynomial functions of degree m = 5.
- The adjusted confidence interval (ACI) was then determined. It was established by using the Student’s distribution.

For each sample, three measurements were carried out to determine the dynamic sorption curves. The number of samples was r = 3; the degree of freedom was v = 2; the observation time was eight hours (480 min), i.e., the number of measured values *n* = 480; the reliability factor was α = 0.1. The adjusted confidence interval (*p* ≥ 90%) was sought.

A detailed description of the statistical analysis is in the dissertation thesis of the author of this article [42].

## 3. Results

The following is a description of the results of all measurements and their analysis.

### 3.1. Averages of Three Measurements

**Figure 8:**
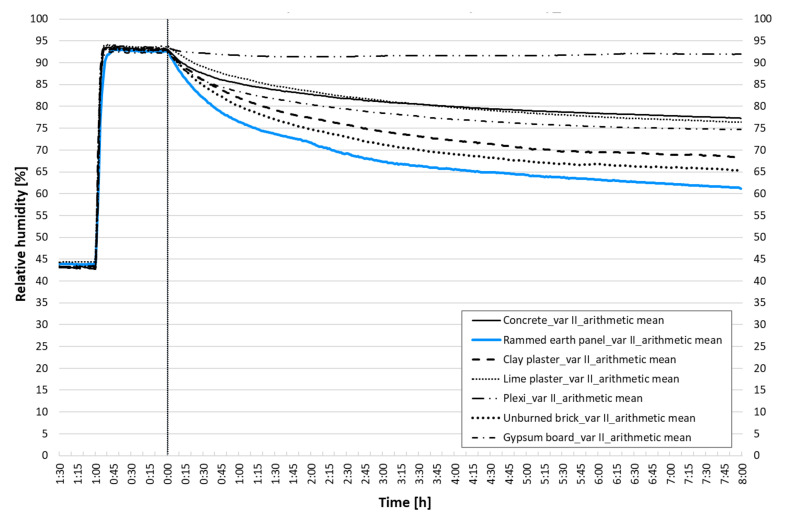
Dynamic adsorption of wall elements: arithmetic mean.

Figure 8 summarizes the results of the performed experiments. Relative humidity courses over time are given as the humidity response of the indoor environment depending on the type of adjacent building structure and its sorption properties. The graph shows the arithmetic averages of the measurements of each wall element in the dynamic adsorption properties experiment.

The structure made of rammed earth clearly has the greatest potential for the adsorption of moisture from the air, followed by unburned brick and clay plaster. The lowest results are shown by nonclay materials. This trend is evident throughout the eight-hour observation period.

After eight hours of observation at a given A/V ratio, the rammed earth panel will reduce the relative humidity in the interior by 34%, unburned brick by about 30%, and clay plaster by 27%. The decrease in relative humidity of nonclay structures after eight hours is 17–20%. The graph also shows the rate of decrease in relative humidity as indicated by the slope of the RH curve. The steeper the slope of the curve, the higher the adsorption rate is. An analysis of the process dynamics is given below.

In the case of rammed earth, the decrease in RH in 60 min is up to 18% (interestingly, this same reduction of RH takes nonclay materials 8 h to achieve). It is also possible to show the time needed to reduce the relative humidity from 95% to 80%: rammed earth panel takes 40 min; unburned brick 60 min; clay plaster 80 min; gypsum board 130 min; concrete and lime plaster approx. 240 min.

**Figure 9:**
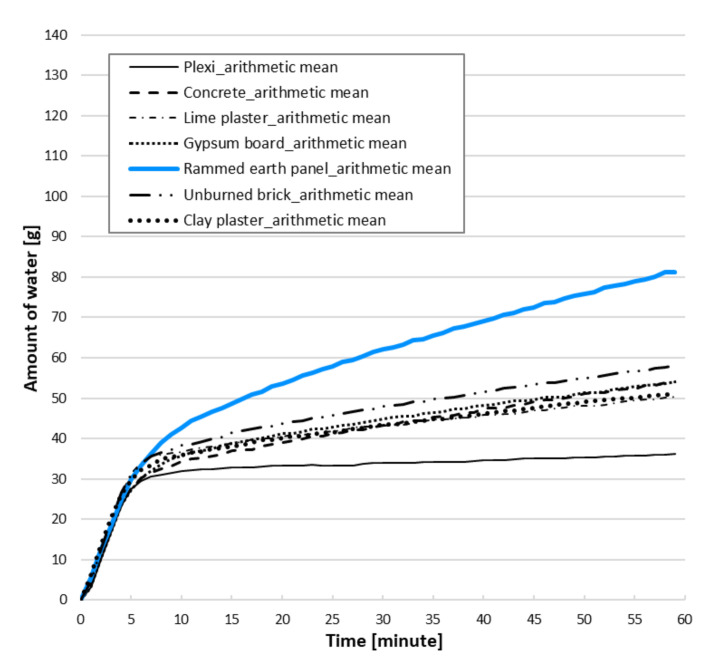
Water consumption during the experiment.

During the experiment, the water consumption required to maintain an RH of 95% for 60 min (humidity gain/source indoors) was also measured. Water consumption curves (Figure 9) show a linear increase from about the 15 min mark, so gradients can also be compared. The slope in the interval of 20 to 60 min is calculated as a linear function *y = ax + b*, where the slope determines the parameter a. According to this parameter (Table 1), the selected structures can be divided into four groups arranged from the smallest to the largest sorption potential: lime and clay plasters; gypsum board and concrete; unburned brick; and rammed earth panel. To maintain the same relative humidity in the chamber, the rammed earth panel consumed 2.7 times more water than clay plaster and other common structures.

### 3.2. Analysis of Dynamic Behaviour

This evaluation of the dynamic experiment was performed because of the slope of the tangent of the regression curve of mean values. This analysis reveals the dynamic potential of the sorption properties.

The slope of the tangent (the first derivative of the regression curve function) was determined at each minute interval. The evaluation was performed for all selected wall elements. The result is the arithmetic mean of each structure.

It should be emphasized that the regression curves do not have a dynamic onset as significant as the measured experimental values. However, it should be noted that the determination of the regression curves was performed according to the same methodology, so that some distortion (reduction of the slope of the tangent in the first minutes of observation) may result from the same error for all structures.

**Figure 10:**
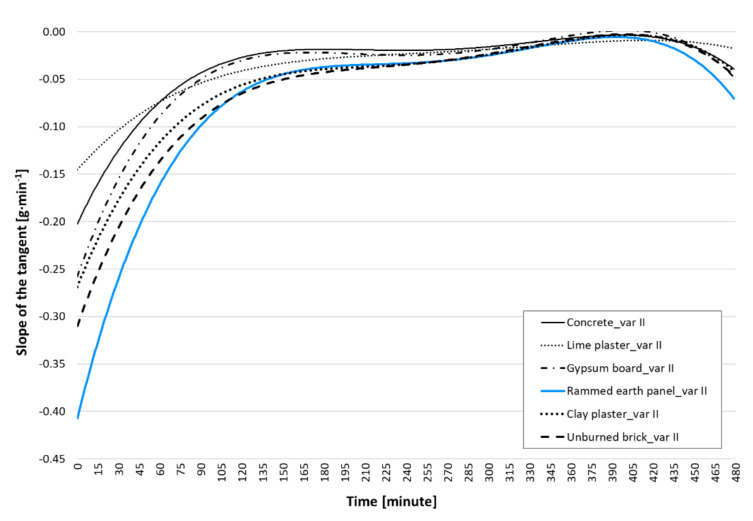
Slope of the dynamic adsorption tangent.

The analysis of the dynamic adsorption behaviour (Figure 10) was divided into five time intervals from the beginning of the observation:

- 0–60 min: The first interval was the most dynamic. Sixty minutes after the start of the observation, the dynamic potential had a half value;
- 60–120 min: In another 60 min, the dynamic potential dropped by another 1/3; this was the last significant dynamic part;
- 120–300 min: The properties of dynamic adsorption of selected materials were grouped as follows: clay structures, concrete and gypsum board, and lime plaster;
- 300–420 min: Identical dynamic properties of all materials. The value of the slope of the tangent was less than 0.017 (conversion to degree < 1 °), i.e., it was almost constant;
- 420–480 min: The values of the slope of the tangent in this interval cannot be used for analysis because the regression curves in this interval allowed for the potential trend of other data (i.e., observations longer than 8 h). The regression curves rose significantly in this interval, even when they did not follow the laws of physics. It would be better to find a more accurate regression curve, but it is extremely time consuming and, for the purposes of this work, this curve is sufficient. Dynamic behaviour is especially important at the beginning of observation.

The evaluation of the slope of the tangent was very useful. This confirmed the expected and measured conclusions about the sorption properties of porous materials in the dynamic state. The highest dynamic potential was verified for the rammed earth panel, followed by clay structures (unburned brick and clay plaster), which were followed, as expected, by commonly used building structures such as gypsum board, concrete, and lime plaster.

### 3.3. Confidence Interval Analysis

**Figure 11:**
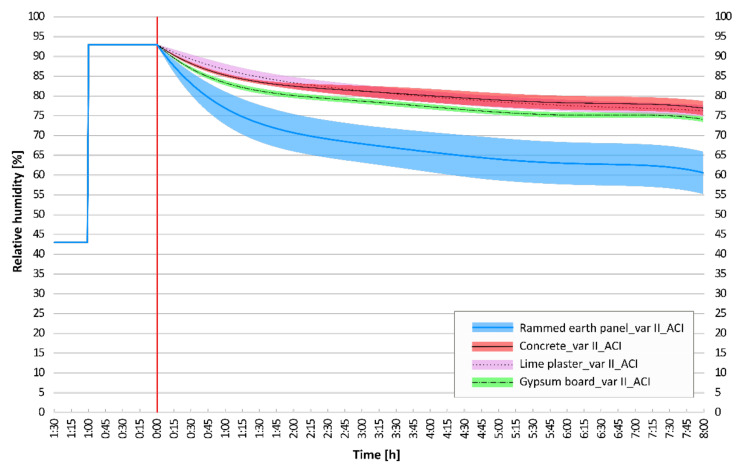
Comparison of adjusted confidence intervals: rammed earth panel, concrete, lime plaster, gypsum board.

**Figure 12:**
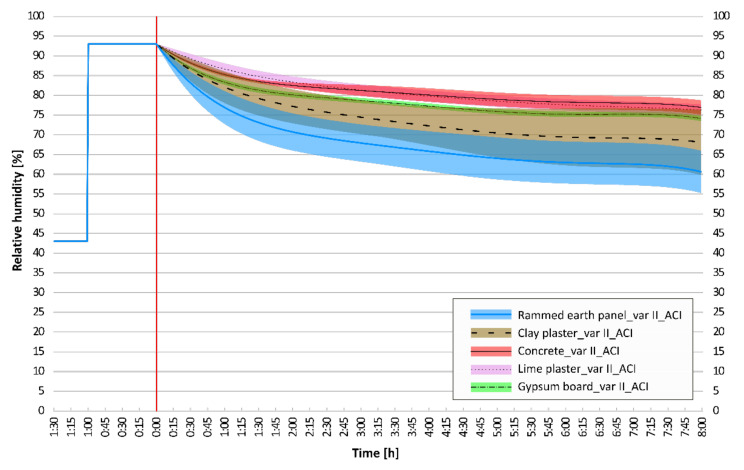
Comparison of adjusted confidence intervals: rammed earth panel, clay plaster, concrete, lime plaster, gypsum board.

**Figure 13:**
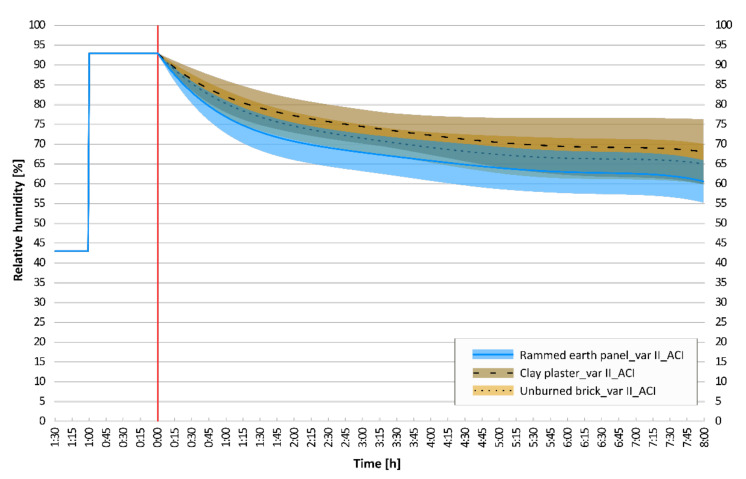
Comparison of adjusted confidence intervals: rammed earth panel, unburned brick, clay plaster.

A graphical comparison of the adjusted confidence intervals for the moisture response results of selected wall elements is essential. This is the main conclusion of the conducted dynamic experiments. The adjusted confidence intervals of the dynamic adsorption experiments, which were performed on six structural compositions: concrete; lime plaster; gypsum board; rammed earth panel; clay plaster; and unburned brick, are shown and compared. The statistical deviations of the regression curve of the mean values are shown in Figure 11, Figure 12 and Figure 13.

Common building structures with homogeneous properties have very narrow confidence intervals. On the contrary, clay structures have inhomogeneous compositions and also inhomogeneous dynamic sorption properties with wider confidence intervals.

When comparing concrete and lime plaster structure, it can be stated that for first 120 min, concrete has a higher sorption potential, and the confidence interval is below the confidence interval of lime plaster. From the 120th minute, the results are almost identical, and the intervals overlap. If the last of the measured typical building materials, gypsum board, is added for comparison, its dynamic sorption properties are even better. Its very narrow confidence interval is just below the lower limit of the confidence interval of previous materials (Figure 11).

If the measurement of the clay plaster was incorrect, this is also a question in this case. However, at the end of the observation, it has a significantly wider confidence interval than other clay materials. The following can be stated: In the worst possible composition of clay plasters, it may happen that the sorption properties of common building materials are comparable to those of clay plasters. In the narrow upper limit of the clay plaster interval, the assumed values of the intervals overlap (Figure 12). However, this condition never occurs in the case of unburned bricks or rammed earth structures.

As expected, when comparing clay materials, the rammed earth panel has the best sorption properties. It can be observed that the confidence intervals overlap, from the upper half of the rammed earth panel to the lower half of the clay plaster. The results show that the sorption potential of the unburned bricks is roughly between that of clay plaster and rammed earth (Figure 13).

The following Table 2 compares the size of the confidence interval at different observation times.

## 4. Conclusions and Discussion

The subject of the research is a detailed description of the sorption properties of materials based on unburned clay. These materials are generally known for their ability to control the relative humidity in the interior.

The process of adsorption and desorption of air moisture into the structure of the porous material is complex. The analysis is more complicated for clay materials because the properties and behaviour of clay minerals also come into play. Therefore, clay structures cannot be easily quantified, and their properties cannot be accurately declared. The resulting clay-based materials are often unique in their properties dependent on the source of the raw materials and its exact mineralogical composition. Therefore, it is difficult to compare the results of experiments across scientific teams if the materials studied have no information on their exact composition.

The results of the experiment show the potential of using unfired clay as an environmentally active material for regulation or stabilization of the internal humidity of the microclimate.

**Lessons from Experiments**

For scientific conclusions, it is important to evaluate experimental data using statistical methods. Such an evaluation may show a different view of the measured and published data.

This is especially important when evaluating data on inhomogeneous materials such as clay structures. These materials have a wide dispersion of the resulting properties, and therefore, it is necessary to take this fact into account and evaluate the data correctly. The variance of the results can be significant depending on the statistical methods used.

**Use of Experimental Data and Continuation of Research**

The results of the medium-dimensional dynamic test can be used for further research and verification of the properties of other designed building structures. It can be used to evaluate the humidity response of the indoor environment to changes in relative humidity when using porous building materials.

In terms of continued research, it would be very useful to create a single-zone moisture transport model that corresponds to the designed and performed dynamic sorption experiments. On the basis of such a model and measured data, the model could be verified and used for more complex modelling of sorption processes. This would help to better understand and describe the complex processes of transport of water vapor in clay materials.

**Sorption Properties and Potential of Clay Materials**

As expected, the sorption properties of rammed earth structures are excellent. Due to production technology, they can contain significantly more clay minerals than other clay structures and thus have the highest sorption potential. Of course, this is also related to the porosity and the specific surface area of the material. The main research was on a structure made of rammed earth developed at the Faculty of Civil Engineering CTU in Prague as a prefabricated load-bearing panel. The sorption properties of similar structures strongly depend on the type and amount of clay minerals used in the structure.

The commercial product of unburned bricks achieved very good sorption results. In all experiments performed, in terms of the quality of the properties, it was just behind the rammed earth structure. The material must have a good source of soil that contains a large amount of clay minerals. A considerable advantage of an unburned brick structure is easier production compared to rammed earth technology and thus lower final cost. Therefore, from an economic point of view, this structure has the greatest influence on the problematic adsorption and desorption of water vapour from the air. The biggest disadvantage, however, is aesthetics. Rammed earth is a much more valuable structure in terms of design. It would therefore be appropriate to expand the market for unburned bricks, but also to focus on their appearance, as this material should be used as unplastered, fair-face brickwork.

The last of the group of clay materials was a detailed examination of clay plaster. They are sometimes overrated for their properties. Although natural clay has excellent sorption properties, in practical use, clay plasters contain a certain amount of sand, primarily to minimize shrinkage. Typical clay plasters contain 60–75% sand, which rapidly decreases sorption ability. Such a high amount of sand means that the sorption properties of clay plaster can be on par with traditional building materials. This is shown by the results of sorption isotherms. However, dynamic experiments have shown that clay plasters still have a greater sorption potential than selected standard nonclay building structures.

However, it is necessary to look at the properties of building structures comprehensively. In addition to excellent sorption properties, clay materials also have low CO_2_ emissions, are suitable for negative ion remediation, and are recyclable, etc. In terms of internal environment and environmental quality, they make very high-quality structures.

**Operating Energy Savings Passive Systems, e.g., by Using Clay Materials**

Compared to the technical solution of indoor microclimate conditioning in the form of forced ventilation, regulation, and intelligent control, environmentally active materials are not comparable to efficiency and, at the same time, are not so dynamic in terms of their control. On the other hand, they are sufficiently robust and immune to extreme situations (black out, failure of the device or its parts, incorrect steering settings, etc.). With an optimal and conceptual building design, these materials can significantly reduce the energy required to operate mechanical indoor environmental quality control systems.

At the same time, this passive solution brings other benefits for the environment—a favorable microclimate; heat and humidity accumulation of massive clay constructions (reduction of heat loss, reduction of the risk of overheating in summer, moisture buffering); discharge of negative ions in the interior; use of natural, low carbon, and easily recyclable materials; etc.

## Figures and Tables

**Table 1:** Slope of the water consumption curve in the interval of 20–60 min.

Tested Structure	Slope of the Curve *a* [g·min^−1^]
Ref: Plexi board	0.07
S1: Concrete	0.39
S2: Lime plaster	0.26
S3: Gypsum board	0.34
S4: Rammed earth panel	0.71
S5: Unburned brick	0.38
S6: Clay plaster	0.28

**Table 2:** The size of the adjusted confidence interval (ACI), in%.

Tested Structure	Observation Time [min]			
	60	120	300	420
Concrete	0.9	1.5	3.5	3.5
Lime plaster	2.9	2.9	2.9	2.9
Gypsum board	1.3	1.3	1.3	1.3
Rammed earth panel	8.4	9.4	10.7	10.7
Unburned brick	8.1	8.6	12.5	15.0
Clay plaster	6.6	6.9	9.5	10.2

## Data Availability

Data available in a publicly accessible repository.
